# Book Reviews

**Published:** 1817-01

**Authors:** 


### CRITICAL ANALYSIS

### *OF RECENT PUBLICATIONS*

IN THE

### DIFFERENT BRANCHES OF PHYSIC, SURGERY, AND MEDICAL PHILOSOPHY.

---

*Elements of Pathology and Therapeutics; being the Outlines of a Work intended to ascertain the Nature, Causes, and most efficacious Modes of Prevention and Cure, of the greater Number of the Diseases incidental to the Human Frame: illustrated by numerous Cases and Dissections.* By CALEB HILLIER PARRY, Member of the College of Physicians of London; Member, and formerly President, of the Royal Medical Society of Edinburgh; one of the Physicians of the General Hospital at Bath, and Physician at the Casualty Hospital, and Puerperal Charity, in that City. Vol. I. General Pathology.—8vo. pp. 463. Printed by Cruttwell, Bath, for Underwood, London.

THE generality of medical writers, it must be admitted, are under the necessity of making themselves known by some means, and can find none so effectual as to write a book. As these early performances are professedly intended for advertisements, they are usually got up with much industry, well written, and only deficient in the most important part—the application of the author's practical knowledge. On this account it is that we hail with unfeigned rapture the production of a veteran, who not only has had time and experience, experience, but has the good sense to turn them to account. If such a writer is a teacher, the only danger is lest, in his elementary arrangement, he should conceive himself obliged to find causes for all the events he has to describe, and should thus labour without materials, and forestal the opinion of his younger readers. We cannot help remarking, that this too often appears in the writing of Dr. G. Foordyce. Dr. Heberden has kept free from these dangers, by confining himself principally to practice; and, when we reflect on the low state of morbid anatomy during the most active period of his professional life, we cannot help admiring his caution, whilst we feel a due sense of gratitude for his invaluable legacy.

Dr. Parry, at a more fortunate æra, has favoured the world with the result of his experience, and with the inductions he has drawn from them through a long series of years. We acknowledge that, when such a work, from such a master, was announced, we raised our expectations, probably, higher than could be gratified; and, perhaps, the work has as much merit as we ought to have expected. The author commences systematically with defining what knowledge is, and what forms medical knowledge.

"All human science is a knowledge of the qualities and order of Phenomena.

"Phenomena may be divided into two classes, single and complex.

"Of the former are the simple Sensations, as a colour, a smell, a sound, &c.

"The latter unite, by apparent propinquity, two or more simple Sensations, as derived through one or more senses. Such are gold, an apple, &c.

"The pictures or recollections of sensations are Ideas; which may be termed Phenomena equally with the sensations which were their prototypes.

"The uniform and inconvertible succession of like phenomena constitutes what is called Cause and Effect.

"The faculty of distinguishing the agreements and differences of phenomena, both as to quality and order, which is necessarily innate, is the whole of human Intuition.

"When, in a great variety of instances, we have observed a like union, or a like succession, of like phenomena, without having been able to discover any exception to this arrangement; if in other cases we see the same congeries or succession, with the exception of only a small number of phenomena, we infer the existence of those which are unseen. This is the true nature of Induction, which is admitted as an adequate basis of rational conclusion."

We cannot, in pathology, admit all that the author requires quires in this last paragraph; or, perhaps, we ought to say, that the postulate should have been illustrated by an example. We should rather say, When we find a constant succession of phenomena, we are entitled to call that succession a LAW till we discover an interruption to that succession; and, in our future inquiries, where we meet with a similar succession of phenomena, with only very slight deviations, it is the business of philosophy to ascertain those deviations, in order to determine how far the law, in other respects, is similar; and to ascertain, if not the cause of, at least the circumstances under which such deviations may be expected.

Dr. Parry next proceeds to a definition of physiology, pathology, and therapeutics, in which it cannot be necessary to follow him. Of the deviations from health, he observes, that the most common is a disordered state of the whole, or of some part, of the sanguiferous system; that is, in the quantity and motion of the blood: the properties of that fluid he defers till he arrives at the notice of particular diseases. Some general remarks follow on the excessive momentum or determination of the blood to particular parts. In the midst of these are incorporated two paragraphs, the inference on which we could have wished had been maintained by facts and reasoning instead of assumption.

"It will readily be admitted, (says Dr. Parry) that all the habits of mankind in civilized society, comparatively with a state of ruder nature, tend to produce an excessive degree of nutrition, and to maintain a proportionable degree of plethora in the human frame.

"This fact affords a presumption as to the prevailing condition of the sanguiferous system in persons under circumstances of moderate affluence; which presumption, taken in connection with more direct proofs of the actually existing state, will contribute to confirm and establish the principle."

All this may be very true, but it is too much to concede at once.

The author proceeds to remark, that the consequence of increased circulation is increased heat, and usually a higher colour of the skin, which, as the blood is the only red substance in the human body, is fairly imputed to the condition and quantity of that fluid on the surface. Some observations follow on the different colour of venous and arterial blood.

The structure and functions of the sanguiferous system are next considered—the structure of the arteries first, and their two coats possessing two powers of motion. In the elastic all authors are agreed. In what Dr. Parry calls the tonicity, there is some difference of opinion. We are not disposed disposed to dispute whether the power of motion, without elasticity, is the effect of muscles or not; but we wish the term *tonicity* had not been introduced, because the power of contraction or of motion would have answered every purpose. It is very true, that Mr. Hunter imputed this power, as well as what he called the sphincter iridis, to muscles; and we are ready to admit, with Dr. Parry, that, as no fibres have been detected by the microscope, nor any fibrine by chemistry, we are not authorised to call the power muscular. We must, however, admit that it has all the properties of a muscle, viz. contraction and relaxation; that, under the latter state, the parts may be elongated; and that the power of contraction ceases with life.

Dr. Parry conceives that the heart is sufficient for all the purposes of impelling the blood through its whole course of circulation. In proof of this, he remarks, that, under certain circumstances, the *saltus*, similar to what is observed in the emission of the blood from a wounded artery, may be seen in a vein. He, however, adds, that, under many circumstances, the vessels of a part take on increase of action without any aid from the heart. From these two facts, we should be led to conclude, that the economy in this, as in almost every other instance, has made a provision beyond what is necessary in the ordinary state of health: that, in common, the blood is propelled to the smaller arteries by the force of the heart; after which, that the powers of the smaller arteries are sufficient to transmit it to the extremities of the veins, and to supply the organs of secretion: that for the latter, probably, a slower circulation may be necessary; but, that when, from any cause whatever, the heart acts with unusual violence, the force extends as far as the veins, inasmuch as the ordinary slow progress, by the power of the smaller arteries, would be insufficient for the safety of the vessels themselves. On the other hand, when the power of the heart is interrupted by the destruction of a large artery, that then the smaller ones increase their action, and possess a power conformable to the necessity of the case. All this is exactly consistent with what is seen every day, in the various conditions of the sanguiferous system. We are, however, obliged to detain the reader for one remark, which we will make as short as is consistent with perspicuity.

Speaking of the power of the heart, Dr. Parry remarks,—

"When, however, a projectile force has once been given to the blood from the impulse of the heart, or of any central part so constructed as to answer the purpose of a heart, we may readily conceive how the usual progressive motion may for some time continue, tinue, after the action of the heart has either ceased, or, at least, has been interrupted with regard to the part.

"To this cause we may, perhaps, in part, attribute not only certain microscopic phenomena observed in the circulation of the blood by Haller and Spallanzani, but also this remarkable fact, that, after death, *all the arteries, whether large or small, are found comparatively void of blood.*

"Although, however, this may be, in part, the cause of the emptiness of the arterial system after death, it is probably only a small part.

"It is more reasonable to conjecture that, in health, arteries are capable of being contracted by their tonicity beyond that degree which would be permitted by their elasticity; but that, after death, the tonicity being shortly lost, the mechanical power of elasticity preponderates, and the artery no longer contracts in proportion to the blood which it contains, and is consequently more or less empty."

The first cause here assigned for *that remarkable fact* that the arteries, whether great or small, are found comparatively empty, is the force of the heart. But, as this force gradually ceases as death approaches, we might rather conclude that the arteries would remain with a considerable quantity of blood in them, which the power of the heart could not forward into the veins. The next cause assigned is, that the elastic power continuing after death, and the tonic power ceasing, the arteries, being more open, are comparatively more empty. But this could not account for the smaller arteries being so empty, because, in them, the elastic power is so comparatively small. What is much more to the purpose is, that, in some modes of dying, the arteries and the left ventricle of the heart are found to contain a very large quantity of blood; and, in most cases, if the ventricles are examined immediately after respiration has ceased, the right side of the heart and the veins will be found with very little blood, and the arteries full. It is surprizing that these facts, so well noticed by the older writers, and so frequently remarked, we may add explained, by Mr. Hunter, are so generally overlooked by modern writers, especially as they form no inconsiderable proofs of death, and often of the mode of dying.

After the more common mode of dying, a body is rarely examined till stiffening has taken place. At the same time that stiffening takes place, the same action is going on in the blood vessels, and by this contraction, whether muscular or not, the arteries are emptied. It was to this, that the arteries owed their name, being supposed by the ancients to contain air or spirit with which they were furnished by the *aspera*

*arteria,*

*arteria*, or trachea. But occasionally, especially in that dreadful form of fever by them called *causus*, the arteries were found full of blood. This induced the methodic sect to consider fever as *error loci*, the blood having, as they conceived, escaped into vessels destined only for the air, or *spiritus*. But it is now found that the phenomenon arises from the sudden death of the whole body, which, in some parts, seems almost to precede the cessation of respiration, since the pulsation of the arteries is imperceptible, and the blood ceases to be oxygenated even while the patient continues to breath. In these cases the body never stiffens, the heart and arteries lose their powers of contraction, and the blood remains in them. In all this, there is nothing contrary to Dr. Parry's position; but we thought it right to account for a want of uniformity in so important a series of actions.

Several paragraphs follow, to prove that the power of the heart, and the tonic power of the arteries, are the effect of life, and that they are excited into action by stimuli or sympathies. This we, as the professed disciples of Mr. Hunter, are not disposed to question. We feel also no inconsiderable satisfaction in perceiving that so accurate a writer as Dr. Parry should probably, from the same necessity, fall into a language often objected to in Mr. Hunter; we mean expressions which might imply rationality, or choice, in certain organs or vessels. Not that such an implication is just. It proves no more, than that in describing certain phenomena, the causes of which we cannot ascertain, we are under the necessity of explaining ourselves by a reference to other phenomena which come the nearest to those we wish to describe. Let the following paragraph serve as an illustration.—Speaking of that part of the blood which is expended in the secretions and excretions, it is added,

"The exertion of these functions themselves affords satisfactory evidence of the tonicity of certain branches of the capillaries, which, though often of equal diameter with others carrying red globules, not only in a state of health refuse to admit them, but select, as it were, what parts of the blood to receive, and what to effuse; and even combine its several molecules, so as to form substances of new and original properties.

"That the capacity of rejection, which exists during health, ceases at some period after death, appears from the various exudations and ecchymoses which we usually see in many depending parts of the dead subject.

"That it also continues for some time after apparent death, is evident; because the matter of coloured injections, which after many hours freely passes into the colourless exhalants, enters them with difficulty at an early period."

The last paragraph is explained by what we observed of the stiffening, which ceases with the contractive power of the arteries.

Several ingenious remarks follow on the various irregularities in the action of the heart, their causes, and the uncertain proofs to be derived from the pulse.* These considerations gradually lead the author to the excessive general momentum of the blood, all which is introductory to the main subject of inflammation.

If excessive dilatation, with increased momentum, arises from temporary causes, it ceases with them.

"On other occasions, the excessive momentum is succeeded by local determinations, which, from their duration or other circumstances, may be considered as morbid.

"The order of succession of local symptoms of this kind, as connected with constitutional ones, is varied chiefly in the three following ways:

"1st. In some instances, the local congestion or dilatation first appears, and is followed by excessive action of the heart, and proportionable general momentum of the blood.

"2dly. In other instances, the local and general momentum seem to occur together, and to proceed with equal steps; and,

"3dly. In other cases, the general increased momentum precedes, and is followed by the local morbid determination."

This statement is followed by a view of the various parts to which the blood may be determined during its excessive momentum, and of the changes or succession of those parts, commonly called the conversion of diseases; but which the author considers only as a change in the part or organ to which the blood is determined. Dr. Parry, with much propriety, declines any enquiry into the causes of this local determination and increased momentum of blood; but, as he professes not to understand the French pathologist on this subject, it cannot be amiss to offer our opinion.

"Bichât speaks of the extraordinary afflux of blood to particular parts, as being the result of the increase of the powers of life. To these words, says our author, I am unable to attach any definite ideas."

This language is not, we believe, entirely peculiar to Bichât. May we not add, too, that, allowing inflammation to be increased vascular action, (which action is the effect of life,) there must be at least a greater vital action than ordinary; and, as we know nothing of life but the actions

* We shall not, at present, make any remarks on a note in which the author conceives that the circulation is assisted by a vacuum in one cavity of the heart; as this we have promised to consider by itself on some future occasion.

it induces, may we not, without any great violation of strict language, call such a condition an increase of life. We are ready to admit that, however small the deviation from accuracy of language may be, we ought to avoid it in philosophical reasoning. We shall, therefore, only consider inflammation as an action of the vessels, greater than what they are capable of in a state of health. Here, then, we are compelled to admit increased power; and, if this increased power is extended over the whole body, it follows, that, in the progress towards such a power of action as constitutes disease, there must be a certain state in which the subject feels in higher health than ordinary: and, that such is the condition of the system previous to most acute diseases, is proved by the experience of the most ancient and wisest physicians. We need only mention one of the earliest and most frequently quoted aphorisms of Hippocrates relative to Athlatæ, and the accurate history of Gout, as given us by Sydenham. We have said thus much, because we shall hereafter endeavour to show that increased vascular action, though one of the phenomena, is not the whole of inflammation; and shall have occasion to remark how many invaluable practical inferences are to be drawn from an attention to the high health which often precedes disease in its most acute form.

The dilatation necessary for the reception of a larger quantity of blood, under increased momentum, Dr. Parry very justly remarks is by no means to be considered the effect of mechanical force from the *vis a tergo* of the heart or larger arteries. In this we perfectly agree with the ingenious author, and are ready to admit that the dilatation frequently, perhaps always, procedes the increased determination of blood to those vessels. This we consider among those properties which an accurate physiologist may trace in every new action which takes place in a living body. It is enough to give a single instance under disease. When matter is formed after acute inflammation, that matter is always determined by the absorption of interstitial parts to the surface; but, long before it arrives at the surface, a blush is seen on the skin, and even an elongation of one part of the cuticle within that blush. This elongation is very properly called *a pointing*, and that place will certainly ulcerate for the escape of the pus, whatever attempts we may make, by an artificial opinion, to evacuate the contents of the abscess. Yet the pointing above described is found to have taken place long before the matter has arrived at the surface; and is exactly analogous to the dilatation of the smaller vessels before before the increased momentum of the blood has extended to them.

Dr. Parry, in our opinion, occupies more time and space than is necessary in discussing the question of proximate causes. If this term must be preserved (and we see no necessity for it), we would confine it to the symptoms. In this case, the proximate cause of the local pain, increased or altered pulse, with other symptoms peculiar to each organ, may be ascribed to those changes in the action and structure of such parts as are not to be seen during life, but which the improvement in morbid anatomy has rendered familiar to us. These changes, therefore, may, we conceive, be called the proximate cause of the symptoms.

Having briefly stated the general progress of increased momentum of the blood, Dr. Parry goes on to show its progress when attended with inflammation, particularly the fluids effused. These effects are slightly enumerated, and some practical and very useful hints, on the cause of renewal of inflammation, usually termed relapse.

We now arrive at an important division of the phenomena of inflammation, according to the nature or texture of the part in which it is seated. In this, according to the late custom of English writers, we find our author following Bichât.

"In the examination of the structure of the animal frame, Bichât has divided the whole into several systems, according to their different textures or functions. These are, the cellular; nervous; vascular, of red and black blood; capillary; exhalant; absorbent; mucous, lining the mouth, stomach, intestines, &c.; serous, forming the pleura, peritonæum, arachnoid, &c.; synovial, lining the cavities of joints, and forming certain sheaths of tendons; glandular; dermoid; epidermoid; pilous; osseous; medullary, forming the marrow; cartilaginous; fibrous, as the periosteum, dura mater, aponeuroses, tendons, ligaments, &c.; fibro-cartilaginous, the ears, alæ nasi, trachea, &c.; muscular."

We cannot help regretting this arrangement, so complicated compared with the simplicity of our own countrymen. Of the membranes, it is enough to make a division of the mucous and those which line cavities. To the term serous membranes we have always objected, inasmuch as; in their healthy state, their secretion is not serum; and to describe a part by its action under disease, is to confound all distinction. If the mode of action in these various parts under inflammation was always different,—most of all, if the treatment were to be different,—all these distinctions might be important; as it is, they appear pedantic or unnecessary. But let us proceed to the author's remarks on these subjects. First, we we are told, that, of the extravasations following inflammation, the most common is serum into the cellular membrane, or into the cavities which the author considers as usually smeared with serum. Several other forms of inflammation are enumerated, under which serum is effused, which, if a healthy process succeed, is afterwards absorbed.

"To what extent (says Dr. P.) serum is, in all cases, capable of being absorbed, it is difficult to say. This capacity is particularly questionable in those cases in which, after inflammation, there is an excess of albumen, or fibrine, or both, either dissolved in the serum, or floating in it in detached portions. I am not acquainted with any facts from which to decide absolutely on this point, though, from the removal of certain hard and painless swellings about tendons, after gout and rheumatism, which we shall again have occasion to mention, one should be inclined to suspect that this might be the case.

"It is more usual that such an effusion terminates in the union of parts that are naturally disjointed, constituting the materials of that process which is called Adhesive inflammation. We every day see this take place from the crusts of that substance, which has usually hitherto been denominated coagulated lymph, effused on the different serous surfaces already specified.

"In this way we may explain, not only the more obvious examples of inflammatory adhesion in the thorax and abdomen, but probably, in some instances, the union of joints by anchylosis, and the permanent rigid swellings of ligamentous and tendinous parts after gouty and other inflammations."

We see no reason for any limitation as to the powers of absorption; and, highly as we respect this ingenious author, we cannot reconcile ourselves to this mode of confounding the various forms of inflammation, especially as we are taught to distinguish them with so much accuracy by that writer whose language Dr. Parry has thought proper to adopt. To what purpose do we distinguish between erysipelatous and adhesive inflammation, if the latter is only an accidental event under the former. In the true adhesive inflammation we find no effusion of serum. Coagulated lymph unites surfaces, which before moved easily on each other, or which were artificially divided. But this is not a mere effusion at the extremities of vessels in consequence of high action. It is a peculiar action, set up at that time, and for that particular purpose. For the same reason we cannot admit, with Dr. P., that the obliteration of arteries, after being wounded, or after their fibrous coats are broken through by ligatures, should be imputed to any thing like inflammation producing extravasation from the exhalants. These processes we consider to be all governed by their own laws; laws; and, that, though the effusion of lymph is among them, the effusion of serum would be altogether inconsistent with, and destructive of, the very ends for which the process is commenced. Still less can we admit, that "the same substances seem to be the materials by which wounded parts unite under the process called union by the first intent." (Page 115.) Union by the first intent, as we have lately had occasion to remark, is inosculation of divided vessels: adhesive inflammation, in the language of the inventor of the term, is union by coagulated lymph only. The *substances*, to repeat Dr. Parry's word in the plural number, never occur in the true adhesive inflammation; and, if adhesion, which is rarely the case, exists during the effusion of serum, the process is always imperfect.

Some very just remarks follow, on the removal of lymph or serum by the process of absorption,—after which we are led to the consideration of pus as a product of inflammation; and here,—as the Hunters have proved by demonstration, and Berzelius and Pearson have both confirmed by chemical experiment, that pus may be secreted without any loss of substance,—we could have wished that this proposition had been admitted without unnecessary discussion.

Inflammation of mucous membranes is next described as sometimes followed by a mere increase of the healthy secretion; at others, the author remarks that he has found the nature of the secretion not distinguishable from pus, and, under very high inflammation, that fibrine or coagulated lymph is effused. In all this it is not Dr. Parry's intention to inform us of any thing new, but to follow the order of his inquiries. The calculous matter which is found in various parts long after effusions of different kinds, is well noticed, with remarks, for the most part satisfactory. At the conclusion of this division, some attention is paid to the condition of the patient, or of the parts under inflammation. These we shall give in the author's words; and with them defer the consideration of the remainder of this interesting performance to a future Number.

"The force and duration of the increased momentum, which are requisite to the production of this morbid effect, greatly vary in different constitutions, and in the same constitution at different times. A considerable degree is necessary in the early and middle periods of life, when all the powers of restoration are strong; and the sum of the two diminishes, *cæteris paribus*, as we reach the term of old age. Hence persons, at the former periods, are more capable of the salutary processes of resolution, adhesion, &c.; while those at the latter period are more subject to have inflammations mations terminate in mortification, or the death of the part, which, if it is a vital one, is immediately followed by that of the whole frame. Where also it is not vital, the constitution, exhausted, as it were, by its own efforts to throw it off, sinks, and the patient dies. Or, if the evil do not reach to this extent, parts so affected are liable to tedious ulcerations, with discharges of a watery or bloody kind, indicative of affections altogether different from those which accompany recovery.

"A state somewhat similar in kind, but different in its progress, is apt to occur in what, with technical barbarism, is called Irritable Inflammation; that is, in habits in which inflammation is easily excited, and, though slight, is readily exchanged for that which is characterized by a state of languor and inactivity.

"It can, I think, scarcely be doubted, that it is this expression of what, in reality, are nearly opposite states in that series of phenomena which is called inflammation, that has given occasion to the assertion of the different microscopical appearances of quickness or slowness of circulation in that malady, either as occurring at different periods of the disease, or as produced by different external causes.

"It may, however, be observed, in passing, that neither the one nor the other state, though demonstrated, should serve as an infallible rule for the administration of remedies; the operation of which is too complex, and difficult of observation, to admit, in our present state of knowledge, of that universality of application, which results from the systems of the schools.

"How far the different states, which have just been described, can be considered as salutary, we cannot, on all occasions, determine. In that of mortification, the local changes are certainly not conducive to the immediate health of the part; which is sacrificed, as something extraneous, and makes way for that which is new and more perfect. So far the process, generally considered, is a salutary one; but the difference consists in this, that in many other circumstances of inflammation, the part restores itself by its own affections, and does not call on the constitution for the fatigue of acting; whereas, in that last mentioned, the part dies, and the constitution is called upon to supply its place. In the other kind of inadequate inflammation already noticed, which is called irritable, there also seems to be a disposition in the constitution to make amends for defective power in the part.

"This law of the constitution, by which it rarely acts when the powers of a diseased part are adequate to the restoration of its own healthy functions, is observable on many important occasions, which will be remarked in the course of this work.

"These are the chief phenomena, and the order in which I have observed them to occur, in Inflammation."

*An Experimental Enquiry into the Effects of Tonics, and other Medicinal Substances, on the Cohesion of the Animal Fibre.* By the late Adair Crawford, M. D. F. R. S. Edited by Alexander Crawford, M. D.—London: Callow and Underwood. pp. 124. 1816.

Few names could prove higher authority for the guarantee of an interesting performance, than that of the celebrated author on the subject of animal heat. The pamphlet is also well-timed, inasmuch as there at present prevails an indolent and mischievous scepticism in respect to the possibility of our arriving at any satisfactory degree of acquaintance with the principles of medicinal agency,—a scepticism partly engendered by the unwarrantable generalization taught in the school of Brunonianism, and too much encouraged by the chylopoietic doctrines of the day,—doctrines in themselves admirable, but faulty and injurious in their extension beyond a certain point. A treatise on tonic medicines wears the aspect of absolute novelty, for some practitioners had almost began to suppose that such medicines had only an ideal existence.

During our perusal of this little volume for the purpose of analysis, two objections suggested themselves to its general tenor: first, the lamented and ingenious author seems to infer too much from the phenomena of inanimate to those of living matter; and, secondly, he now and then substitutes hypothetical assumptions for the data supplied by cautious and close reasoning. With these exceptions, however, we have found every thing to be satisfied and pleased with in the tract now before us.

"It is upwards of twenty years (says the respectable editor) since the following treatise was prepared for the press by the author, but the publication of it was prevented by his death, which happened a year afterwards. It is unnecessary to trouble the public with all the reasons that have delayed the publication since, only for the last six years; the time it has been in my possession, a very infirm state of health prevented my attending to any serious pursuit. The copy from which this is printed is in my brother's hand-writing, and, however it may be received by those who are most interested in such investigations, I believe it will not be thought presumption to allege, that it bears marks of that industry, ingenuity, and just reasoning, which, in the opinion of the philosophic world, distinguished the former writings of the author."—Preface, page 1.

Dr. Crawford informs us, that he was led to undertake the experiments detailed in the present work from the circumstance of his having observed, in the course of his experiments ments on the matter of cancer, the changes which the fibres of animals underwent by immersion in the poison of cancer in contact with common air, changes which he supposes referrible to the action of a peculiar fluid, denominated by him animal hepatic air.

"As this fluid (he says) abounds in nature, being found wherever the putrefaction of animal substances exists, it seemed not improbable that many of the morbid appearances in the human body might be ascribed to its influence. For it is manifest, that whatever has a tendency to destroy the cohesion of the fibre, must, if not counteracted, eventually give rise to disease and to death."

Hence, we suppose, he would infer the lax fibre and generally lower standard of health observable in individuals who inhabit crowded cities, compared with the hard flesh and robust constitution of the labouring rustic.

Dr. Crawford imagines the different conditions of the animal fibre to be capable of the following divisions and definitions.

"By the term *cohesion*, (he says,) I mean to express, not only the power inherent in bodies which resists the disunion of their particles, but, likewise, that which prevents the particles from changing their relative positions, and from yielding to such forces as have a tendency to separate them to a greater distance from each other. Hence, it will appear, that under the cohesion of the fibre, I comprehend its firmness, elasticity, and strength, affixing to those terms the following significations.

"By the firmness of the fibre, I mean to express the force with which it resists *impression;* by the elasticity, its power of resisting *extension*, and of restoring itself when the extending cause is removed; by its strength, the force which it is capable of exerting in opposition to such causes as tend to destroy the continuity of its parts.

"The first of these properties is known by the touch; the second, by the comparative extensions which the fibres undergo, when they are stretched by equal small weights; and the third, or the *strength* of the fibre, is known by the weight which is required to break it."

"Those substances (he goes on to say) which diminish the firmness and elasticity of the fibre, I shall call relaxants; those which increase its elasticity, tonics; and, those which increase its strength, corroborants. It is proper to observe, that, in the living animal, the tone of the fibre is a compound effect. It depends not only on the elasticity of the simple solid, but likewise, as my learned colleague, Dr. Fordyce, has justly observed, upon the energy of the vital principle; in consequence of which, the approximation of particles during life is greater than that which would be produced by their elasticity alone. The tendency to approximation, which they derive from this cause may, I think, properly be expressed by the term *contractility.* And hence, in the living animal, those substances must be considered as tonics, which increase the contractility, as well as the elastic force of the fibre."—P. 2-4.

Having premised these considerations, Dr. Crawford goes on to detail a series of experiments which he made upon animal fibre; first, by subjecting it to the influence of *vinous and spirituous liquors;* secondly, of *narcotics;* thirdly, *vegetable bitters;* fourthly, *acids and alkalies;* fifthly, *neutral and earthy salts;* and, lastly, *metallic preparations:* and, in the course of describing these experiments, he intersperses several physiological and pathological remarks, which are stamped throughout with his wonted and characteristic ingenuity.

How far those inferences are just in reference to medicinal agency on the *living* system *generally*, which are formed from the application of such substances only to a *small part* of *dead* matter, may admit of a question; for our own parts, as above hinted, we think that the deductions of our author are sometimes hardly strict enough as to this particular.

"With a view (says Dr. C.) to determine the changes which the fibre might undergo by exposing it to the action of port wine, six portions of the small intestines of a kitten, were taken, each of which was two inches and a quarter in length. Three of these were introduced into a phial which was nearly filled with port wine, and closed with a cork; and the remaining three were immersed in water as a standard. Being placed in a cool situation during three days, the portions in contact with the wine were found to have greater firmness than those that were immersed in the water. The sum of the weights required to break the former was 9lb.; to break the latter, 7lb. 4 oz. Each of the portions immersed in the wine being stretched by a weight of six ounces, the sum of the extensions was six degrees nearly. That of the standard, in similar circumstances, was twelve degrees."

After describing a similar experiment made with sherry wine, which proved that this last is rather more operative in the way of strengthening the fibre than the port wine, Dr. C. proceeds to state the following trial made upon living animals.

"I took two kittens of the same litter, as nearly equal in bulk as possible; they were about a fortnight old. To one of them I gave two tea-spoonfuls of port wine, mixed with an equal quantity of milk. To the other, I gave as much pure milk. At the expiration of an hour they were drowned. The stomach of the kitten that had swallowed the wine was then separated from the duodenum and the œsophagus; and, an incision being made along the shortest line from the cardia to the pylorus, the coagulum of milk and wine was removed; the stomach was laid upon a flat piece of cork, and was divided longitudinally into two equal parts; these were then cut by the double knife (an instrument which Dr. C. contrived for the purpose) in such a manner that each of the portions should be one-tenth of an inch broad. The sum of the weights required to break them was 24 oz. The same experiments being made with similar portions of the stomach of the other kitten. The weights required to break them were 19 oz. The sum of the extent of the former, when each of the pieces was stretched by a weight of six ounces was one-tenth of an inch; of the latter, one inch."

Our author next proceeds to make experiments on the effects of vinous and spirituous liquors upon the *skin*, by which he finds that, in this case, contrary to what takes place in the intestinal fibre, the cohesion of this part (the skin) is diminished, and, from such contrariety of operation, he argues in the following manner.

"Since, therefore, it is proved, that wine increases the strength of the stomach in a living, as well as in a dead, animal; and, since it is found, that in a dead animal it diminishes the cohesion of the skin, is it not probable, that when it is mixed with the blood, and carried by the circulation to the surface, it will produce a similar effect upon the skin of a living animal."

"If this be admitted (Dr. C. goes on to say) it will, I apprehend, throw some light upon the mode of operation of wine, when it is employed as a medicine. There is, I think, no doubt, that vinous liquors principally act upon the nervous system. They appear, when taken in moderate quantity, to excite the energy of the brain, and to increase the activity of the vital principle. But, besides these effects, we learn from the preceding experiments, that wine augments the tone and vigour of the stomach, and at the same time relaxes and debilitates the skin. Does it not, by these properties, determine from the center to the surface of the body? And hence, does it not, when judiciously administered, assist in preventing morbid affections from falling upon parts which are essential to life, and conspire with the efforts of nature in throwing off what is injurious by the pores of the skin?"

The above reasoning seems to partake of the humerol pathology, as well as of the older notions of the methodic system of physic which talked of *constricted*, and *relaxed*, and *mixed* state of fibres; this, however, ought not to lessen respect, could we satisfy ourselves, that, in point of fact, Dr. Crawford is quite correct in assuming, that wine, at the same time that it augments the tone of the stomach, necessarily relaxes the skin; at any rate, it does not do it with that regularity and certainty that it ought to do, were the above hypothesis of its *modus operandi* capable of being substantiated. And, when its administration is so conducted as to insure its sudorific agency, there is often nothing of a morbid nature to be thrown off by the pores of the skin, even even allowing to these organs, the power of elaborating and ejecting this same matter.

Opium is the next material of our author's experiments, and the results show, in this instance also, that the cohesion of the intestines is increased, while the skin and nerves are relaxed and debilitated by this drug. Dr. C. introduces into this division, practical hints on the operations of opium, the utility of which cannot be questioned, however questionable may be the theory on which they are grounded. Opium ought, as well as alcohol, to prove universally a sudorific, were the analogical inference of our author correct as to its probable mode of influencing the system; whereas, the fact is, that one of the most formidable objections to the administration of opium is often the constringing effect which it has upon the skin, as well as upon the intestines.

The exhibition of this medicine (opium) is always most proper and least likely to be attended with mischief when the pores of the skin are open, as is beautifully illustrated by a case in Dr. Currie's work on fever; but it will there be seen, that it was necessary to produce the perspirable state of the surface by other means, while opium was administered; the mere administration of the drug, without such other means, not being adequate to a sudorific effect.

There are facts connected with the relative and sympathetic actions of the stomach and outer skin, which require to be much more attended to and methodized than they are at present, to furnish any satisfactory materials, before we can venture to generalize. Dr. C. has, however, found the clue to the proper method of investigation, and it will behove other pathologists to pursue those researches which he had so ably begun, when Death arrested the hand of the experimenter, and closed the reflections of the philosopher.

We are sorry that our limits will not permit us to detail the trials made with the Peruvian bark and vegetable bitters, which are the materials employed next to the narcotics. This substance (the Peruvian bark) Dr. C. found, like opium, to have a different and opposite effect upon the intestines, blood vessels, and nerves, from that which it occasioned upon the skin, increasing the cohesion of the former, and diminishing that of the latter.

"It appears (says he) from the foregoing experiments, that the bitters which are most commonly used in medicine, increase the strength of the intestines in the following order: Peruvian bark, galls, camomile flowers, gentian root, colombo root, cascarilla, myrrh, and serpentaria.

"It is proper to observe, that galls produce a much greater constriction in the intestine than the Peruvian bark. They render it firmer to the touch, and less extensible by small weights.

 It

It would seem, therefore, that they act more forcibly as a tonic, although they have, probably, less power as a corroborant. But, what constitutes the principal distinction between them is, *the difference of their action upon the skin.* For the one increases the strength of this part of the animal body in a very considerable degree, while the other acts powerfully upon it as a relaxant and corroborant."

Why, we would ask, upon this theory is it, that "the bark in large doses proves a very efficacious remedy in the cynanche maligna, and in the erysipelatous inflammations which appear in the London hospitals?" These are both diseases of surfaces, and both diseases in which tone, rather than relaxation, is required to be given to such surfaces. Indeed, our ingenious author seems in some measure to overlook his reverse sympathetic theory, if we may so term it, when he speaks in another place of bark being proper in such and such diseases, inasmuch as "it impresses a degree of tone upon the stomach which can be borne without injury, *and which may be communicated with safety to other parts of the system.*"

Acids and alkalies were both found in the experiments "to diminish the cohesion of the fibre." How does this consist with the acknowledged tonic power of the one, and occasionally, perhaps, of the other set of substances?

In the course of our author's experiments on saline materials, he finds that common salt (muriate of soda) greatly increases the cohesion of all the soft parts of the animal body; and, hence, (he says) probably the reason why this salt, which is furnished in such abundance to the inhabitants of the earth, is so admirably calculated to preserve and to restore health. He finds, however, that this substance (salt) occasions a much greater augmentation of strength in the arteries than in the veins, and, from this peculiarity, he infers, the virtue of salt in scrofula, inasmuch as it gives an impulse to those vessels (the arteries) which supply the pabulum of glandular secretion. Even allowing these conclusions to be somewhat fanciful, how much more pleasing are they to witness than that indolent dogmatism which can find nothing but *general* strength, or *general* weakness, in any of the diseases to which the human frame is subject.

The very fashionable and very important salt, magnesia vitriolata, *(sulphas magnesiæ,)* our author finds "possesses an extraordinary power of strengthening the intestines," and reasons upon this power in the following manner.

"Hence, probably, the reason why this salt, in small doses, frequently repeated, is one of the most efficacious remedies hitherto discovered in constipations arising from inflammation in the bowels.

In

In such cases it is known, that a stricture takes place in the inflamed portion of the intestine, and that the portion immediately above the stricture is distended by the fæces, which are carried downward in consequence of the peristaltic motion. This distension is counteracted, partly by the energy of the vital principle, and partly by the cohesive force of the intestine, assisted by the action of the abdominal muscles. If these forces be insufficient to resist the distending power of the fæces, the superior portion of the canal will be stretched beyond its tone. But it seems to be a law of the animal economy, that, when the stomach or intestinal tube is stimulated by a distending power, or by any other cause, to a certain degree, the peristaltic motion is inverted, and vomiting is produced.

"It is plain, that, in these circumstances, salutary effects must arise from the exhibition of a cooling laxative, which has the property of communicating a sudden increase of tone and of strength to the alimentary canal. For, in that case, the mischief arising from the distension will be counteracted; the cause which immediately gave rise to the inversion of the peristaltic motion will be removed, the canal will be enabled to propel its contents downwards with greater force; and, consequently, its power of overcoming the stricture will be increased.

"Upon similar principles we may, I think, explain the reason why a stricture of the intestine is frequently removed by the application of cold fomentation to the abdomen."

We shall make no other remark on the above suggestions of our author in reference to the modus operandi of a much used medicine, than to say, that the observations, whether ill or well grounded, may prove serviceable in drawing the attention of the prescriber to the principles of medicinal operation, as opposed to the practice of mere empirical routine. If any particular medicine has done good, we are not to wait to know the *quo modo* of its agency before we repeat its application, but it would prove greatly to the interests of medicine as a science, were the theory of healing agency less disregarded in the practice of the healing art.

It is in this view, indeed, that we are more particularly disposed to set a value upon the labours of Dr. Crawford; and we take occasion to urge, upon the medical student especially, the necessity of experimental investigation, as well as a mere observation, of unconnected facts, in order to enable him to practise medicine with conscientious feelings and happy effect.

We are sorry that our limits only allow us to state the bare results of the remaining experiments of Dr. Crawford.

Glauber salts *(sulphas sodæ,)* had very trifling effect either upon the intestines or the skin. Neither was there much effect produced by sal. ammoniæ, *(murias ammoniæ.)* The several mercurial preparations tried by our author, had the effect of increasing the cohesion of the fibre generally; tartarized antimony "increases the cohesion of the intestines, and diminishes that of the skin." Nitrate of silver rather diminished cohesion; and, contrary to our authors expectation, he found the same diminution from the sulphas ferri, the sulphas cupri, and sulphas zinci. These effects, being contrary to his anticipations, Dr. C. attempts to account for by the acid which enters into the composition of the several materials employed; but we are not satisfied with his reasoning in this instance, since the salt formed by the union of an acid and metal is very different in its actual nature and physical effects from either of the ingredients solely considered. But we must here reluctantly take leave of our ingenious experimentalist, with feelings of the highest respect for his talents and industry, and with our sincere acknowledgments to the editor for bringing the last lucubrations of his esteemed relative before the eye of the public.

---

*Medico-Chirurgical Transactions, published by the Medical and Chirurgical Society of London.* Vol. VII. Part I.—Longman and Co. 1816.

(*Continued from vol.* 36, *p.* 489.)

*An Account of the last Illness and Death of Professor H. Benedict de Saussure;* by Louis Odier, M.D. Professor of Medicine in the Academy of Geneva; and one of the Foreign Members of the Society.

In giving our opinion of this case, we shall commence with the examination of the body, and afterwards connect the appearances with the symptoms. In doing this, contrary to the order as we find it, we shall begin with the viscera of the trunk, as the symptoms of disease appeared earliest in them.

"The abdomen presented a singular conformation: for, when the integuments and peritoneum were opened and turned back, so as to expose the viscera, neither the stomach, the liver, nor the colon could be seen, nothing but small intestines and cæcum presenting themselves. This last was of enormous size. Its circumference measured fifteen inches, while that of the ileum at its termination in the cæcum was not more than four or five inches. The appendix vermiformis was four or five inches long, and adhered by its extremity to the posterior surface of the cæcum. The colon, entirely hid by the small intestines, was also much dilated, and ascended to the right side, over the liver, to the diaphragm as far as the sixth or seventh rib, then passed to the left side, where it reached as high as the fifth rib, immediately under the nipple, so that in this place it was nearly contiguous to the apex of the heart, being being separated from it only by the diaphragm, which it had pushed considerably upwards. Thus, the colon, instead of passing as usual below the liver and the stomach, passed above these viscera. It bestrode the stomach at its superior orifice, and compressed it so much the more, as, instead of descending as in the natural state on the left side, it passed down obliquely to the right side, so that its sigmoid flexure was contiguous to the cæcum: at length it terminated in the rectum, which was also dilated to such a degree, as to equal the ordinary size of the colon. All the other abdominal viscera were sound. There was no trace of obstruction, hardness, or other morbid affection of the pancreas.

"With regard to the viscera of the thorax, the lungs were small, but in other respects healthy, as were also the heart and the large vessels. But as the diaphragm was pushed considerably upwards, so as to admit of very little room for the expansion of the lungs in respiration, if the dimensions of the thorax had been confined within its ordinary limits; this was compensated for by the lungs being extended under the clavicles to some height along the neck. We had not sufficient time to examine minutely the attachments of the pleura and mediastinum in this singular conformation."

In turning back to the history, we are equally disappointed and surprised to find scarcely a hint to direct us as to the time of life at which each of the complaints commenced. We are only told, that the professor enjoyed, early in life, a state of health and strength which promised an hereditary longevity; and that, "from the age of fifteen he had been liable to hæmorrhoids, which occasionally bled profusely."

"His health had been uniformly good, till the time when he took a voyage to the Boramean Islands, about thirty years ago; on which occasion, having eaten a large quantity of acid fruits, he was seized with a long and serious disorder, which was supposed to have its seat in the stomach, or in the pancreas, and which rendered him incapable of retaining any species of nourishment. All remedies were ineffectual, excepting Starkey's soap, which was of great service to him. Ever after the period of this illness, he was subject to symptoms of deranged digestion, was tormented with flatulence, and with so inveterate a disposition to generate acid in the stomach, that, although he lived for a great number of years almost entirely upon meat, and other animal food, and avoided, with the greatest care, fruit, green vegetables, and acid liquors, he regularly experienced, for some hours after dinner every day, a kind of pyrosis or burning sensation, which obliged him to have recourse to chalk, or other absorbents, and to reject, by vomiting, a great part of what he had eaten. He had fortunately acquired the power of thus emptying the stomach without any particular inconvenience. If he, at any time, endeavoured to resist this urgent call to vomit, he used to feel a great sense of weight pressing on his stomach, accompanied by strong acidity, and a species of anxiety so oppressive, that nothing could assuage it but immediate mediate vomiting, to which he was always at last obliged to have recourse. In general, however, he did not wait till the distress had attained this degree of severity; and he chose so well his opportunity for relieving himself, that most of his friends, and even his relations, never knew that he was subject to this infirmity."

We pass over more minute circumstances, unconnected with the organic derangement, to allow room for Dr. Odier's reflections on the above.

"If (says he) we consider the state of the abdominal viscera, I may remark that the displacement of the colon, and the compression that it exercised on the superior orifice of the stomach, present a very natural explanation of the habitual vomitings which the patient experienced some hours after his meals. The extreme dilatation of the large intestines may have been the consequence of the great appetite, which his frequent mountain excursions tended to create; especially as he was in the habit of satisfying it by food that was coarse, and calculated to afford a large quantity of fæces, which the exercises he took would tend to accumulate, and to harden in the track of the large intestines. I had long ago been convinced of the dilatation of the rectum, from its producing symptoms of compression on the vesiculæ seminales, which the patient had often mentioned to me, and which I could explain on no other supposition. This circumstance also, perhaps, contributed to the incontinence of urine, which had tormented him for the last months of his life; but what I certainly could not foresee was, the displacement of the colon. I do not pretend to decide whether this displacement was coeval with birth, or was the gradual effect of dilatation. I am, however, inclined to the latter opinion. I must at the same time acknowledge that an accidental displacement of the colon in this direction, is a phenomenon which I have never witnessed, is unprecedented in the records of medicine, and is apparently in opposition to the natural tendency of the gravity of the contents of this intestine, which would be that of depressing its curvature and of drawing it down towards the pubis, instead of pressing it against the diaphragm. It must, however, be considered that the great muscular strength of De Saussure, and the continual exercise which had become habitual to him, especially that of ascending steep acclivities, in which the abdominal muscles are in strong action, would contribute powerfully to increase their tension, and would tend to overbalance this effect of gravity, and to force the intestine upwards by the obstacle opposed to its descent.

"Lastly, the diminished capacity of the lungs might be supposed to explain the affection of respiration which Mr. De Saussure experienced on very high mountains; but this cannot be admitted as the sole cause, since other philosophers, whose chests were amply capacious, suffered greater inconvenience from the rarefaction of the air at much less considerable elevations. The symptoms which he describes as having felt in these exalted regions, are, besides, nearly the same as those which are stated by the French academicians who ascended the Cordilleras: and there is no likelihood that any similar organic defect existed in them, for I have never seen any other example of the same kind. It is still, however, remarkable that it produced no sensible effect on his habitual state; and there is, perhaps, still more difficulty in understanding how the immense quantity of fæces which must have passed through the colon so near to the heart, had never interfered in the least with its action."

Every reader will join with us in the value of the above as a pathological record. We shall now give the examination of the brain, and connect it with the previous symptoms.

"The body was opened thirty-two hours after death. The dura mater was strongly adhering, particularly along the longitudinal sinus. Between the pia mater and arachnoid coat, we found a considerable effusion of a gelatinous substance, similar to what is often met with in the brain of persons who have died of comatose affections. It had the same bluish tint, peculiar to that substance; excepting that here and there circular spots of a different colour were observed, being of a greyish yellow, and about three or four lines in diameter. They appeared as if encrusted within the membranes, and breaking down into small detached spheres, each of which was surrounded by a small circular border of a blackish red colour. We took these spots, at first sight, for hydatids; but when we attempted to separate them from the membranes, their red margins were found to be blood-vessels, connected with the other vessels of the head; and contorted, from what cause I know not, into circles. There were no separate pouches, or solution of continuity in the membranes; only in these places they were more transparent than in others, and the serosity which was underneath communicated freely with that which was spread over the whole surface of the brain, being of the same nature and of the same colour, namely yellowish. This colour was apparent in these places on account of the transparency of the membranes, whose opacity in the rest of the surface give it a bluer tint. From whichever of these places the membranes were opened, the serosity flowed out like water, and we thus collected about two or three table-spoonfuls. We heated it by the flame of a candle: there was no coagulation, but a strong ebullition with large bubbles; and the whole fluid evaporated without leaving any sensible residue.

"The effusion had taken place, not only on all the surface of the cerebrum, but also on that of the cerebellum, in which latter it was much more considerable on the right than on the left side. We here and there perceived a few air bubbles, mixed with the blood, in some of the blood-vessels.

"The ventricles were also entirely filled with the same kind of serosity, and in such quantity as to dilate them considerably. We estimated that, on the whole, there was about five ounces. The choroid plexus appeared to be almost entirely composed of clusters of hydatids; an appearance which is indeed very common, depending altogether on the dilatation of the very delicate vessels which form the plexus, and not on detached hydatids. The pineal gland was hard, and crumbled like earth between the fingers; but this is also no uncommon appearance. The examination of the head presented nothing further worthy of notice, except that the brain was a good deal flattened at the temples, and deeply furrowed by the arteries."

"At the close of the year 1793, after a long continuance of painful exertions in endeavouring to stem the torrent of our political revolutions, and much domestic anxiety, he was suddenly attacked with vertigo, followed by a distinct feeling of numbness in the left arm and leg: a feeling which nothing could ever remove, though the vertigo was not of long duration. It was to no purpose that I had recourse to blistering, purgatives, frictions with flannel, and with mustard; followed by a long catalogue of antispasmodic and tonic remedies. This affection of the limbs appeared to be seated more in the sentient extremities of the nerves, than in the moving fibres. The arm executed with facility every kind of movement, but conveyed no distinct sensation of touch. He felt always as if a quantity of sand were interposed between his fingers and the objects to which he applied them. This sensation was even to a certain degree painful and agonizing; as if the chief morbid affection of the hand had consisted in an excess of sensibility, so that he was afraid to use it without the protection of a glove. A sensation somewhat analogous to this was also felt in the cheek and the mouth of the same side, which, when he passed his hand across his face, made him sensible of a line of demarcation, very distinctly defined between the right and left side; a sensation which was extremely disagreeable. In other respects he enjoyed good health, and shewed no symptom of plethora, or of debility. He also retained for a long time, his wonted presence of mind, and the full vigour of his intellectual faculties. Many months elapsed without the least change, although this interval was occupied with trials of a multitude of remedies. The cold bath, the hot bath, electricity, arnica, valerian, blistering, embrocations, travelling, hot mineral baths, both artificial and natural, such as those of Aix, Bourbon, and Plombieres; vegetable juices, changes of diet, &c. all were ineffectual. The disorder grew worse, but almost always by sudden accessions more or less distinct and severe. One of the most violent of these seizures occurred at the baths of Bourbon, and was brought on by a *douche*, that had been made too hot; and so complete was the attack, that the whole of the left side, from the foot to the tongue, was affected by it. His speech became gradually confused, and almost unintelligible. His legs, especially the left one, were raised or bent with difficulty. This was most perceptible when he attempted to walk in a straight line, following the junctions in the floor of his apartment, a kind of exercise, in which, from constant practice, he had become very expert, having pursued

 it it with the view of accustoming himself to tread with security on the narrowest ledges of mountains, and borders of precipices. But his disorder had now deprived him of the power of preserving his balance, and his limbs were no longer obedient to the determinations of his will. The most remarkable circumstance, however, attending this state, was, that after he had become so infirm as to require the assistance of a stick in walking, it was in going in or out at the door of a room, that he experienced the greatest difficulty. He could cross the room with a tolerably firm step; but the moment he reached the door, although open on both sides, and leaving a space much wider than his body, and without any difference of level in the floor, it was a most arduous undertaking for him to pass through. He tottered and precipitated his motions, as if he were preparing for the most perilous leap; no sooner was the difficulty surmounted, than he recovered his former confidence, and proceeded with ease across the passage, until he came to another door, when the same unaccountable terrors again assailed him, and the same caution and trouble were requisite to achieve the steps by which the invisible barrier was to be passed. On his return to Plombieres, he had a copious herpetic eruption on the forehead and about the eyes: it was hoped that this would relieve his other complaints. A similar expectation was entertained from the effects of a hæmorrhoidal colic, which suddenly attacked him; but all these hopes were delusive. The disease continued to gain ground."

Blisters produced a temporary relief, and the use of oxygen air proved so highly diuretic as to induce a suspicion of diabetes, which suspicion was much encreased by an immoderate thirst, voracious appetite, and acidity at the stomach. The urine, however, afforded no sugar, though the patient had lived on vegetable diet from the time that his paralysis was confirmed. His vomiting had ceased from the commencement of the paralytic symptoms.

"The disorder (continues the narrator,) advanced rapidly, though by very insensible gradations; the powers of intellect became impaired; he was hardly able to walk; his features sunk, and his body became more and more bent to the left side. He fell into a sort of apathy, from which he only occasionally revived, and for the moment was only capable of taking a part in conversation. He laboured for a time under incontinence of urine, and next had a spasmodic contraction in three fingers of the left hand; and then a gangrenous ulcer on the prepuce. From these complicated infirmities he was soon delivered, by a tranquil, and somewhat sudden dissolution. He had supped on the preceding evening with good appetite, but had been restless in the night. In the morning he turned his head on one side, and breathed his last without a struggle."

The following are the author's reflexions on this part of the examination:

"It appears that the proximate cause of the disease and death of Professor de Saussure, was the effusion of a great quantity of serosity in the ventricles, and between the membranes of the brain; which effusion produced a great compression, and consequent injury of the functions of that organ. I presume it had commenced between the membranes of the cerebellum, because it was only in that situation that we found any difference between the right and left sides: and the disease, though general towards its close, had been for a long time confined to the left side, which, as is well known, leads to a presumption that the right side of the brain was the most affected. An effusion similar to the one above described is a very frequent cause of apoplexy, but in general it takes place suddenly, and the disease lasts only a few days. I have lately seen a gentleman of 61 years of age attacked with apoplexy, which proved fatal in sixty hours, whose head presented on dissection exactly the same appearances as in the case of De Saussure, in whom the disease was of five years' duration. It is singular that so great a derangement in the organization of the brain, and existing for so long a period, should have occasioned so little alteration in the intellectual powers; another singular circumstance is that, with the exception of some transient dimness of sight, the eyes had never been affected; and that the pupil had always contracted readily. The usual consequence of effusion in the ventricles producing the hydrocephalus of children, is a dilatation of the pupil; and it may be understood how in the case of Mr. De Saussure, the disease having increased only very gradually, the optic nerves might have accustomed themselves to a degree of compression, which, had it been sudden, would have affected them; but in the illness of the gentleman above-mentioned, which lasted only three days, no dilatation of the pupil was observed. On what does this difference depend? It will probably be long before this question can be determined. Affections of the brain to all appearance perfectly identical often produce very different effects, and, conversely, effects that are perfectly similar arise frequently from very different morbid affections. In most cases we are equally in the dark with regard to the cause of the effusion. But in this instance we may reasonably attribute it to the cares, the anxieties, and the struggles, with which the mind of our illustrious countryman had been harassed by the eventful political revolutions above alluded to. On the other hand, I have learned since his death that, at the beginning of 1793, he had met with a severe fall down a stone staircase, a cause which, it is well known, often lays the foundation of hydrocephalus; but his son has assured me, that, long before this period, his father frequently mistook one word for another, without being conscious of his mistake, so that he was irritated at not being understood, a circumstance which would appear to indicate that his illness should be dated prior to the first attack in 1793. Lastly, it may also be conceived that the displacement of the intestines may, to a certain extent, have compressed the great vessels, and thus have impeded the freedom of circulation in the head."

Here

Here we think it our duty to detain our reader for a few minutes. We shall say nothing of the last cause, to which the author doubts whether the affusion of the brain may be attributed. It is of a piece with the late Dr. Fothergill's opinion concerning the compression of the *cava* by a hearty supper, and argues so very contracted a view of the resources and provisions of the economy as to deserve no notice.

It is more to the purpose to remark, that the deceased was of an athletic constitution; that he was plethoric from the early climacteric period of puberty; and that, later in life, he was incapable of that hard exercise which might be best suited to such an economy. That the disease in the brain was the effect of effusion or inflammation, or both, is very plain; and also that paroxysms of the above occurred at different times. "The disorder," we are told, "grew worse, but almost always by accession, more or less sudden or severe." The last, and, probably, all the others, were preceded by apparently higher health than usual. "He supped," we are told, "on the preceding evening with good appetite;" was restless in the night; and died in the morning.

When extravasation on the brain takes place in young subjects, it is a remark of the ablest practitioners that the patient rarely escapes without a permanent loss of the use of a limb or of a side. In older people, if they live, all the parts more commonly recover. From a very frequent examination of such subjects, we do not scruple to impute this difference to a cause similar to what we remarked in tracheitis in young and older subjects. When inflammation, or even when mere extravasation, occurs in young subjects, the effused lymph coagulates, and retains, not only its life, but strong powers of organization. Hence the coagulum is frequently found with vessels communicating with the neighbouring parts; and, when such is the case, though all the other effused substances, whether serum or blood, may be absorbed, and any other injury the brain may have suffered be repaired; yet the organized substance will remain, having a capacity to support itself; and, by remaining, either press upon or occupy part of the substance of the brain, and thus impede the proper communication of that part with the corresponding members. In older subjects, there is less danger of the coagulum forming vessels to support itself: hence the whole extravasated part may be absorbed, and the brain recover its functions. On the ingenuous remarks concerning the sensibility of the ophthalmic nerves, and the great uncertainty of the effects induced by the various affections of the brain, we shall refrain to make any animadversions; versions; acknowledging, with the author, our ignorance of these subjects, and regretting, as we doubt not he will do, the coarse treatment which Spurzheim has received in these islands. Whatever may have been his errors, should the man who has thrown the smallest ray of light on this obscure part of physiology be treated with disrespect, not to say with vulgarity and rudeness?

Attached to this paper are some additional remarks by Dr. Marcet. These remarks contain the history of Professor Ferguson's case, who, at the age of 50, was attacked with hæmiplegia, from which he recovered, and lived to the age of 93. For a man of such animal powers, it is true, 50 is not a very advanced age; but it should be remarked, that, at the period of his seizure, instead of high health, he had made a fatiguing excursion into the country, in very cold weather, and returned to town, just before dinner, very much chilled. In this condition, he ate and drank as usual; was seized with hæmiplegia; and, fortunately, *immediately* bled and purged. By an abstemious diet, he perfectly recovered; that is, the extravasated fluid was absorbed, and the functions of the brain preserved.

*(To be concluded in our next.)*

---

